# A new analytical workflow using HPLC with drift-tube ion-mobility quadrupole time-of-flight/mass spectrometry for the detection of drug-related metabolites in plants

**DOI:** 10.1007/s00216-020-02429-7

**Published:** 2020-01-22

**Authors:** Franz Mlynek, Markus Himmelsbach, Wolfgang Buchberger, Christian W. Klampfl

**Affiliations:** Institute of Analytical Chemistry, Johannes Kepler University Linz, Altenberger Strasse 69, 4040 Linz, Austria

**Keywords:** Plant metabolism, Pharmaceuticals, Diclofenac, Drift-tube ion-mobility mass spectrometry, Environmental analysis

## Abstract

Investigations into the interaction of xenobiotics with plants (and in particular edible plants) have gained substantial interest, as water scarcity due to climate-change-related droughts requires the more frequent use of reclaimed wastewaters for irrigation in agriculture. Non-steroidal anti-inflammatory drugs are common contaminants found in wastewater treatment plant effluents. For this reason, the interaction of nine edible plants with diclofenac (DCF), a widely used representative of this group of drugs, was investigated. For this purpose, plants were hydroponically grown in a medium containing DCF. For the detection of unknown DCF-related metabolites formed in the plant upon uptake of the parent drug‚ a new workflow based on the use of HPLC coupled to drift-tube ion-mobility quadrupole time-of-flight/mass spectrometry (DTIM QTOF-MS) was developed. Thereby‚ for chromatographic peaks eluting from the HPLC, drift times were recorded, and analytes were subsequently fragmented in the DTIM QTOF-MS to provide significant fragments. All information available (retention times, drift times, fragment spectra, accurate mass) was finally combined‚ allowing the suggestion of molecular formulas for 30 DCF-related metabolites formed in the plant, whereby 23 of them were not yet known from the literature.

## Introduction

In March 2019 the European Commission (EC) released a Communication (COM (2019) 128 final) titled “European Union strategic approach to pharmaceuticals in the environment” [1]. Therein, an emerging problem regarding the pollution of soil and water bodies due to increased usage of pharmaceuticals is spotted. Several routes for pharmaceuticals entering the environment are possible. These include amongst others the spreading of manure from treated animals on acreage, effluents from fish farming, or (when focusing on drugs for treating humans) drug residues from effluents of wastewater treatment plants (WWTP), or still improper disposal of unused pharmaceuticals via the toilet. The problem of drug residues in WWTP effluents was addressed in the urban wastewater treatment directive of the EC, which has been continually updated since its release in 1991 [2]. Although WWTPs use several steps for the clean-up of wastewater, namely a pretreatment, a primary, a secondary and/or a tertiary/advanced treatment, contaminants may not be removed completely and can subsequently be found in the effluent [3–12]. This is of particular concern when the treated wastewater is re-used, e.g., for irrigation in agriculture, an approach that has become common practice in many arid regions [13]. Thereby, drug residues can be taken up by the crops [14–17] and subsequently translocated from the roots to other plant parts, whereby drug-related metabolites may be formed as has been revealed by a series of studies [18–20].

Non-steroidal anti-inflammatory drugs (NSAIDs) are widely used, resulting in their almost ubiquitous presence in the aquatic environment [21, 22]. For that reason, this group of compounds plays an important role in investigations on the interaction of plants with pharmaceuticals upon uptake from either water or soil. Uptake and metabolization of NSAIDs and in particular diclofenac (DCF) by plants has been the subject of several studies so far [23–30]. The most common approach for investigating translocation and metabolization of drugs in plants is the treatment of model plants with relatively high concentration (high μg L^−1^ to mg L^−1^ range) of the parent drug (or a mixture of drugs) and subsequent analysis of the plant extracts. For the (tentative) identification of new drug metabolites, plant extracts are analyzed by high-performance liquid chromatography (HPLC) coupled to high-resolution mass spectrometry (HR-MS) or high-resolution dual-stage mass spectrometry (HR-MS/MS) [18]. Recently, in two studies, focusing on the detection of statin-derived metabolites in cress plants [31] and the metabolization of sunscreen ingredients in duckweed and *Cyperus alternifolius* [32], HPLC hyphenated to a drift-tube ion-mobility quadrupole time-of-flight/mass spectrometer (DTIM QTOF-MS) was presented, introducing ion-mobility as a further dimension of analysis to this field.

In this work, we present a new “reverse-engineering” workflow facilitating the discovery of new drug-related metabolites formed in plants using HPLC coupled to DTIM QTOF-MS/MS detection. Combining data from chromatography (retention times) with those from QTOF-MS/MS (accurate mass, fragmentation pattern) and DTIM (drift times, collision cross sections (^DT^CCS_N2_)) can particularly enhance the correct assignment of chromatographic/mass spectrometric signals to proposed structures. Especially the compound-specific drift time (DT) was used for the tentative identification of unknown drug-related metabolites. This strategy has been tested on the example of a series of edible plants (categorized by their carbon fixation pathway), namely maize, millet, amaranth, and sorghum (C4 type), and tomato, onion, salad, rice, and pea (C3 type) grown hydroponically and treated with DCF. Thereby tentative formulas for a series of 30 DCF-related metabolites formed by the plants upon uptake of the parent drug from an aqueous growing medium could be suggested, 23 of them not yet known from literature and described for the first time as a result of the new “reverse-engineering” workflow.

## Experimental

### Materials and methods

Diclofenac sodium salt and formic acid ACS (≥ 96%) were purchased from Sigma-Aldrich (Steinheim, Germany). Acetonitrile (ACN), hydrochloric acid (37%) and methanol (MeOH) were supplied by VWR Chemicals (Vienna, Austria). Ultrapure water was taken from a Milli-Q water purification system (Millipore, Bedford, MA, USA).

A 10000 mg L^−1^ stock solution of diclofenac was prepared in MeOH und further diluted in tap water for plant irrigation. This high concentration of the stock solution was selected in order to add a maximum volume of 200 μL MeOH to 100 mL tap water (20 mg DCF L^−1^) in the growing experiments, thereby avoiding damage to the plants by methanol.

### Instrumentation

A 1260 Infinity II HPLC system consisting of a 1260 Flexible Pump, a 1260 autosampler, and a 1290 MCT (all from Agilent Technologies, Waldbronn, Germany) equipped with an Agilent InfinityLab Poroshell 120 Bonus-RP column (3.0 × 100 mm, 2.7 μm) combined with a C18 Guard column (4 × 3.0 mm, Phenomenex) was employed. The injection volume was set to 25 μL. As detector an Agilent Technologies 6560 DTIM QTOF-MS equipped with a Dual Agilent Jet Stream Electrospray Ionization (Dual AJS-ESI) source and a gas kit (Alternate Gas Kit, Agilent Technologies) was used. The Dual AJS-ESI was operated in positive mode. Nitrogen was used as drying gas and sheath gas. The drying gas temperature and the sheath gas temperature was 275 °C, both with a flow rate of 10 L min^−1^. The nebulizer gas pressure was 50 psi, the capillary voltage 3500 V, the nozzle voltage was 1000 V, and the fragmentor voltage was 400 V.

The mobile phase consisted of (A) ultrapure water with 0.1% formic acid (v/v) and (B) acetonitrile with 0.1% formic acid (v/v). The gradient elution was operated as follows: 20% B between 0 and 0.5 min, linear increase to 95% B between 0.5 and 11 min, holding 95% B for 2 min, and re-conditioning with 20% B for 5 min.

The instrument was auto-tuned in the 2-GHz extended dynamic range setting in the 1700 m/z fragile ion mode. The IM trap fill time was set to 10000 μs with a trap release time of 300 μs. The frame rate was 0.9 frames sec^−1^, the IM transient rate was 18 transients frame^−1^ and the maximum DT was 60 ms. For DT measurements used for ^DT^CCS_N2_ calculations, a single field calibration had to be done with the Agilent Tune Mix prior to the sample measurement. The IM parameters for DT measurements used for ^DT^CCS_N2_ calculations were as follows: 1567-V drift tube entrance, 217-V drift tube exit, 210.5-V rear funnel entrance, 38-V rear exit funnel (taken from [33]). These four parameters were fixed in the acquisition method independent of the tune parameters.

### Germination of seeds and growing of plantlets

The seeds of tomato, salad, onion, rice, amaranth, and millet were germinated in Petri dishes containing filter paper soaked with drug-containing water (0 or 20 mg DCF L^−1^). The Petri dishes were sealed with Parafilm® and the seeds were germinated for 7 days in the dark at room temperature.

The seeds of maize, pea and sorghum were soaked in water overnight. They were further germinated on wetted kitchen roll for 2 days and then transferred into a container filled with tap water and ironing beads as plant support. After further 4 days of growing, the plantlets were transferred into Erlenmeyer flasks filled with drug-containing water (0 or 20 mg DCF L^−1^). The plantlets were harvested after seven more days of growing.

### Harvesting, extraction, and analysis of plantlets and germinated seeds

The grown plantlets or germinated seeds were rinsed with water and patted dry with kitchen roll. The plantlets were divided into a root and an upper part. No differentiation regarding plant parts was done in the case of germinated seeds. One gram of plant material (wet weight) was weighed in 15-mL centrifugation tubes. One milliliter of acetonitrile and 2 mL of 0.1 M HCl were added and the plant parts were homogenized with an Ultra-Turrax (Type TP18/10, Janke&Kunkel IKA-Labortechnik, Staufen, Germany). The extracts were centrifuged for 10 min and filtered through 0.45-μm Rotilabo® syringe nylon filters (Roth, Karlsruhe, Germany) into 1.5-mL HPLC glass vials and stored at − 80 °C until analysis.

For the determination of possible metabolites, the samples were analyzed with an HPLC DTIM QTOF-MS. From the experience of earlier experiments, it was known that the highest concentrations of DCF and its related metabolites were found in the roots of the plants. Therefore, only the root extracts were used for further analysis.

### Data evaluation

For data evaluation, Agilent MassHunter Qualitative Analysis B.07.00, IM-MS Reprocessor and IM-MS Browser B.10.00 were used.

IM data of the root extracts were first recalibrated with the IM-MS Reprocessor and were then calibrated with the recorded single field tune using IM-MS Browser. ^DT^CCS_N2_ values were determined using feature extraction (IMFE) in the IM-MS Brower with the following settings: Chromatographic processing of “common organic molecules” with a limited charge state of *z* ≤ 3. The ion intensity was set to ≥ 100 and the retention time was restricted to 2–10 min.

## Results and discussion

### Novel “reverse-engineering” workflow for the detection of unknown drug-related metabolites

The new “reverse-engineering” workflow is based on the two-dimensional separation of compounds by HPLC and in the drift tube of an ion-mobility instrument, followed by the subsequent fragmentation of all the ions in a collision cell. The combination of HPLC retention time and DTIM drift time allows the visualization and investigation of conjugates originating from one certain molecule of interest faster and with higher specificity than with HPLC alone. DCF was selected as the model compound and the approach was tested with nine different edible plants, categorized by the carbon fixation pathway (C3 or C4).

When conjugates of a chemical compound are fragmented in a tandem MS experiment, instrumental conditions can be set up in a way leading to a specific fragment for this group of compounds. In our case of DCF-related metabolites measured in the positive mode, the most obvious and distinct fragment is the DCF ion (m/z 296.0240) itself. For our approach, we have set up a HPLC DTIM QTOF-MS/MS experiment, where compounds are separated by HPLC, ionized using a Jet Stream ESI ion source and are subsequently separated according to their collisional cross section in the drift tube. After the drift tube, all ions are fragmented in a collision cell and finally detected by the TOF mass analyzer. DCF metabolites tend to cleave off parts of the molecule which were attached during metabolism even at low collision energies. In our workflow it is very important, that the ion is fragmented with enough energy to yield the characteristic fragment, while retaining the information of the intact precursor. Even for large DCF conjugates with glucose (Glc) and malonic acid (Mal) (e.g., DCF-Glc-Glc-Mal-Mal) a relatively low collision energy setting of 5 V is enough to produce the DCF fragment with an m/z 296.0240 as well as the precursor ion. For small DCF conjugates on the other hand (e.g., DCF-Glc-Mal), the collision energy setting of 5 V also produces the characteristic fragment but does not completely destroy the precursor.

When the ion trace of m/z 296.0240 is extracted from the HPLC-MS chromatogram, peaks will appear, whenever a compound yielded the distinct DCF fragment that is looked for. Because of the high-resolution instrument, the ion trace can be extracted quite narrow and information about compounds containing the DCF backbone is specific. So, in this first step, a general identification of signals originating from compounds that contain DCF is obtained. The next step is the assignment of the precursor ion which produced this fragment. This can be challenging, because one is dealing with a plant extract and several compounds eluting at the same time can be expected. When instruments without ion mobility are used, the only way to achieve this assignment is trying to align HPLC retention times of all signals, which can sometimes be misleading in complex samples. In the present workflow with the DTIM QTOF-MS/MS, another specific parameter is available, because the ions pass the drift tube as intact molecules and are separated according to their collisional cross section resulting in compound-specific DT. This means, all fragments, which originate from one precursor, are produced after the drift tube and therefore appear with the exact same DT. So, the original precursor ion, which produced the certain fragment, in our case DCF, shows the highest m/z at the given DT and retention time combination. It can thereby easily be distinguished from MS signals from plant matrix (not associated with DCF) as their DT values at a given retention time are different. A proper HPLC separation is still the important basis of our workflow like it is for typical MS^E^ approaches, but combining it with DTIM allows us to assign species easier, faster and even more specific.

**Fig. 1:**
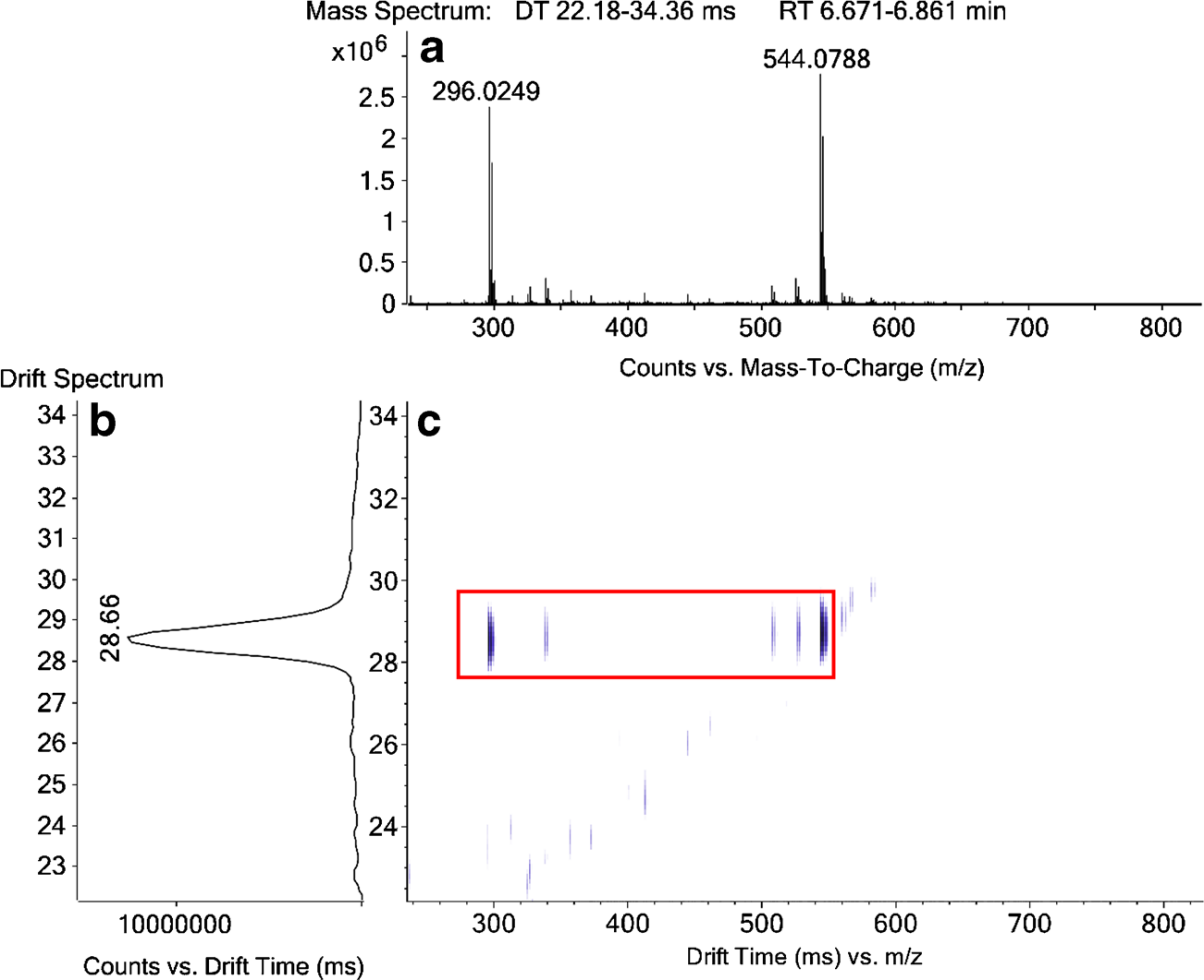
MS and DTIM data for a peak at 6.8 min in an onion sample. (a) shows the mass spectrum from 6.67 to 6.86 min, (b) the drift spectrum for the selected range of 22.2 to 34.4 ms, and (c) the corresponding DT versus m/z plot. The peak at 6.8 min was found to be DCF-Glc-Mal

As an example, the evaluation process is shown in the following for a highly abundant diclofenac metabolite in an onion sample. The mass spectrum and the corresponding ion mobility DTs for all HPLC peaks can be extracted separately. Figure 1 (A) shows the mass spectrum, (B) the drift spectrum and (C) the DT versus m/z plot for a peak at 6.8 min. When evaluating (C), it can be identified quickly which m/z are linked via the same DT (moved through the drift tube intact and fragmented after the drift tube) and which signals elute at the same retention time but are not connected to the DCF fragment via the same DT. The starting point is the spot for the DCF fragment at DT 28.66 ms and m/z 296.0249 in Fig. 1 (C). One can move along the m/z axis to higher m/z values and evaluate which spots are connected via the same DT of 28.66 ms (red box in Fig. 1). The largest m/z which resulted in a DCF fragment is m/z 544.0788, so this is the intact metabolite that should be assigned. When evaluating the mass spectrum in Fig. 1 (A), several low abundant signals between m/z 296.0249 and m/z 544.0788 can be identified, but with the additional DTIM information, one can see from the DT versus m/z plot (C), that only three of these MS signals actually belong to the DCF metabolite. Most signals have significantly lower DT and are therefore not connected to the DCF metabolite. In the case of this DCF metabolite, signals corresponding to two water losses of the metabolite and a [DCF + C_2_H_2_O + H]^+^ fragment can be seen. When evaluating the mass difference between 296.0249 and 544.0788 (Δm/z = 248.0539) the metabolite can be tentatively assigned as DCF-Glc-Mal.

Employing this workflow, eight metabolites could be suggested which contain the DCF structure as a stable backbone. From literature, it is known that DCF forms three phase I metabolites, namely diclofenac-OH (DCF-OH), diclofenac-lactam (DCF-Lac) and diclofenac-lactam-OH (DCF-Lac-OH) [34–36]. These three structures also represent stable backbones of DCF-related metabolites and the acquired data can be analyzed in the same way as it was done in the case of the DCF structure.

**Fig. 2:**
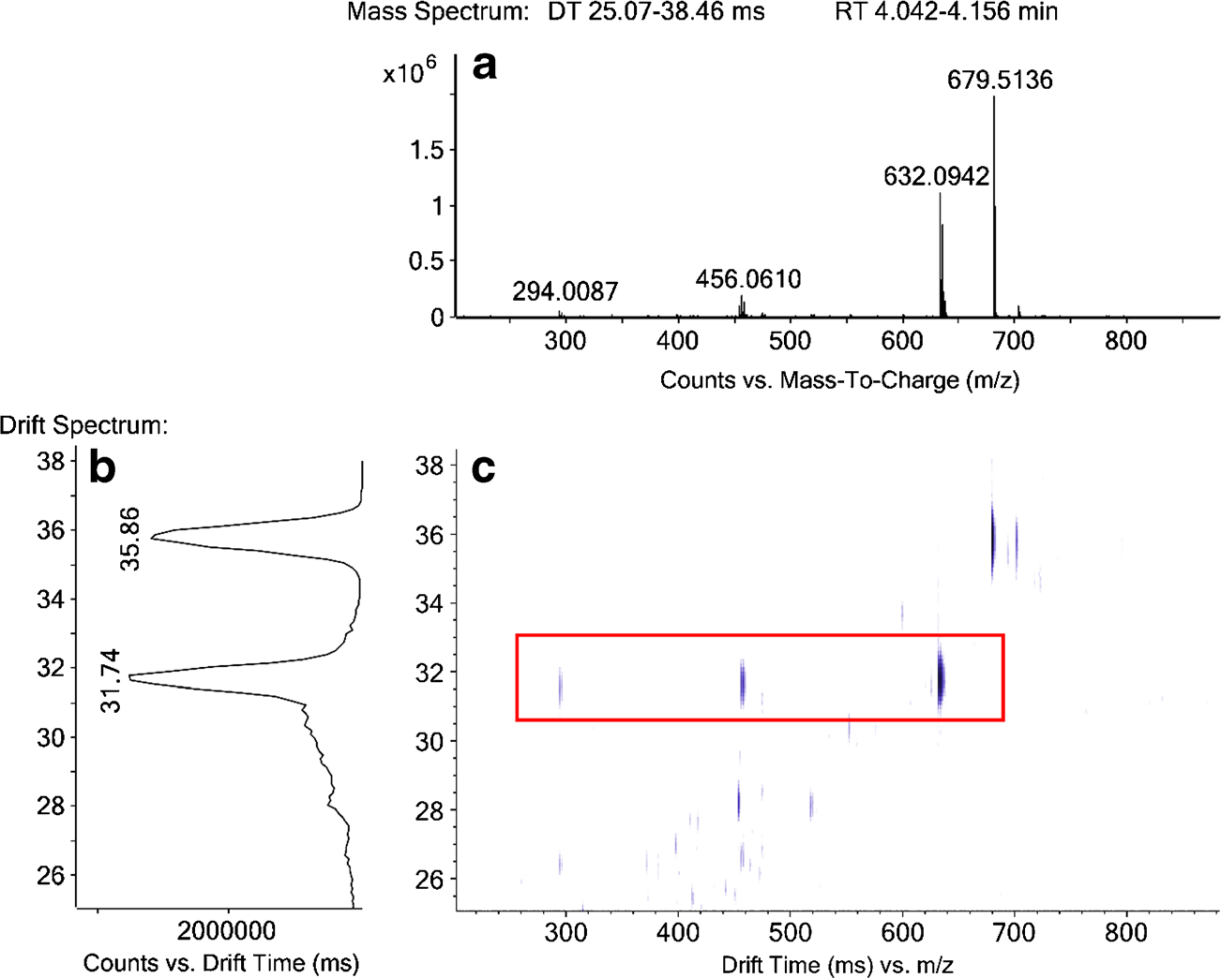
MS and DTIM data for a peak at 4.1 min in a maize sample. (a) shows the mass spectrum from retention time 4.04 to 4.16 min, (b) the drift spectrum for the selected range of 25.1 to 38.5 ms, and (c) the corresponding DT versus m/z plot. The peak at 4.1 min was found to be DCF-Lac-OH-Glc-GlcA

**Fig. 3:**
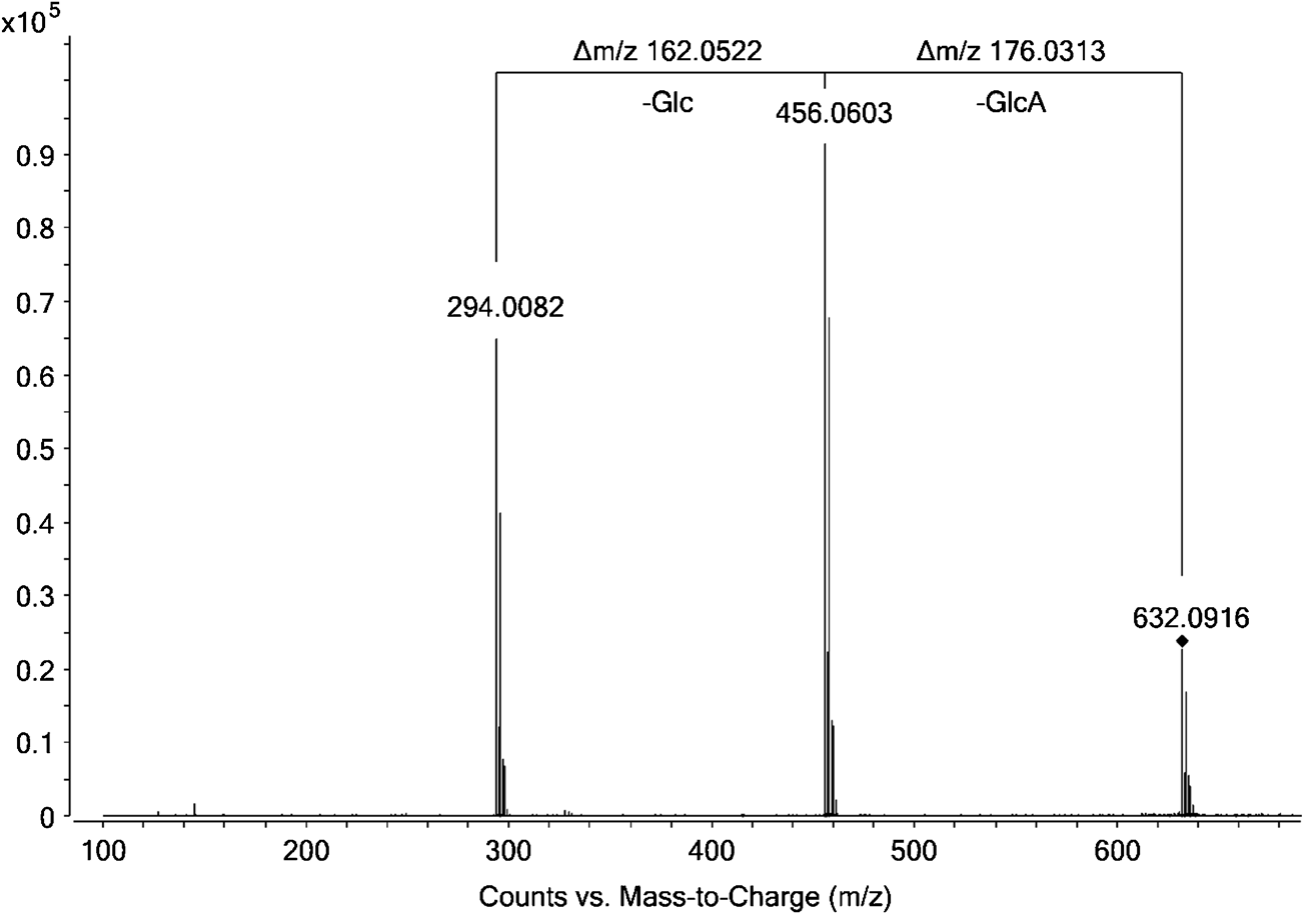
MS/MS spectrum of DCF-Lac-OH-Glc-GlcA (m/z 632.09) in a maize sample fragmented with a collision energy of 10 V

Figure 2 (A) shows the mass spectrum, (B) the drift spectrum and (C) the DT versus m/z plot for a peak at 4.1 min in a maize sample. In the mass spectrum (A) the characteristic signal for DCF-Lac-OH (m/z 294.0087) can be identified. When evaluating (C), it can be assigned which m/z are linked to this DCF-Lac-OH backbone via the same DT (m/z 294.0087, m/z 456.0610, and m/z 632.0942 in the red box) and which signals elute at the same retention time but are not connected to the DCF-Lac-OH fragment via the DT of 31.74 ms. This example clearly demonstrates the benefits of using the DTIM QTOF-MS/MS. When looking at the mass spectrum (A), m/z 679.5136 is the most abundant signal at that retention time. But when including the DTIM information from the DT versus m/z plot (C), it becomes immediately apparent that this signal does not belong to the DCF metabolite, since it has a different DT. The largest, and in this case most important, m/z which resulted in a DCF-Lac-OH fragment therefore is m/z 632.0942, so this is the intact metabolite that should be assigned. The signals between m/z 294.0087 and m/z 632.0942 with the same DT are used for the suggestion of a potential molecular structure. Clearly visible are the neutral losses of 176.0332 (glucuronic acid, GlcA) and 162.0523 (sugar moiety), which allow to assign this metabolite to the structure DCF-Lac-OH-Glc-GlcA. To confirm the correct assignment of fragments, a spectrum from a MS/MS experiment is shown in Fig. 3, where m/z 632.09 was isolated as a precursor and fragmented with a higher collision energy of 10 V.

### Detection of drug-related metabolites in nine edible plants

Nine edible plants, namely maize, millet, amaranth, and sorghum (C4 type) and tomato, onion, salad, rice and pea (C3 type) were grown hydroponically in water containing DCF. Subsequently, the plants were harvested, extracted and the extracts analyzed with respect to the presence of drug-related metabolites employing the workflow described above. The summary of the results can be seen in Table 1. All root extracts were measured in triplicates. Based on accurate mass measurements, MS/MS measurements and a plausibility check using published data from comparable studies, a series of 30 DCF-related metabolites formed by the plant upon uptake of the parent drug from an aqueous growing medium could be identified tentatively. Thereby the parent drug and five metabolites could be detected in all plants investigated. Comparing these findings with the literature published so far reveals that 23 of the tentative metabolites listed in Table 1 have not been described so far in context with plants treated with DCF. This may also be attributed to the novel workflow for metabolite detection presented in this work. Out of these transformation products, two are particularly exceptional. So far, metabolites formed through glucuronidation are primarily known from animal studies [37]. There is only a single paper (published very recently) reporting (based on HPLC- MS data) the formation of glucuronic acid adducts in meadow plants treated with monopatel, a veterinary anthelmintic [37]. We were able to detect four DCF-based substances, where our data suggested a high probability for the presence of metabolites containing glucuronic acid. Two obviously isomeric structures (DCF-OH-Glc-GlcA) with identical mass but slightly different ^DT^CCS_N2_ values and retention times were detected in maize. Furthermore, DCF-Glc-GlcA was found in amaranth and DCF-Lac-OH-Glc-GlcA again in maize. An MS/MS spectrum showing the fragmentation of the latter substance is depicted in Fig. 3.

**Table 1:** Overview of tentative formulas for DCF phase I and II metabolites found in various root extracts of C3 and C4 plants

Name	Formula	RT	m/z [M+H]^+^	delta m/z ppm	CCS (Å^2^)	RSD CCS %	n	Salad C3	Tomato C3	Onion C3	Millet C4	Sorghum C4	Maize C4	Pea C3	Amaranth C4	Rice C3
DCF-OH-Glc-Glc-Mal-Mal	C_32_H_35_Cl_2_NO_19_	4.9	808.1252	− 0.1	264.5	0.5	9	Yes	×	Yes	×	×	×	×	Ye	×
DCF-Glc-Glc-Mal-Mal	C_32_H_35_Cl_2_NO_18_	6.3	792.1314	1.2	250.5	0.3	3	×	×	Yes	×	×	×	×	×	×
DCF-OH-Glc-Glc-Mal	C_29_H_33_Cl_2_NO_16_	5.4	722.1257	1.2	249.4	0.3	3	×	Yes	×	×	×	×	×	×	×
DCF-OH-Glc-Glc-Mal	C_29_H_33_Cl_2_NO_16_	5.3	722.1253	0.6	258.7	0.1	3	×	×	×	×	×	×	×	×	Yes
DCF-OH-Glc-Glc-Mal	C_29_H_33_Cl_2_NO_16_	4.1	722.1250	0.1	255.8	0.6	21	Yes	Yes	Yes	×	Yes	Yes	×	Yes	Yes
DCF-Glc-GlcA-Mal	C_29_H_31_Cl_2_NO_16_	6.0	720.1100	1.0	248.2	0.0	3	×	×	×	×	×	×	×	Yes	×
DCF-Glc-Glc-Mal	C_29_H_33_Cl_2_NO_15_	5.8	706.1302	0.2	243.9	0.2	12	×	Yes	Yes	×	Yes	×	×	×	Yes
DCF-Glc-Glc-Mal	C_29_H_33_Cl_2_NO_15_	6.4	706.1295	− 0.7	245.7	0.1	3	×	×	×	×	Yes	×	×	×	×
DCF-Glc-Glc-Mal	C_29_H_33_Cl_2_NO_15_	6.3	706.1291	− 1.3	253.6	0.2	2	×	×	×	×	×	Yes	×	×	×
DCF-Lac-OH-Glc-Glc-Mal	C_29_H_31_Cl_2_NO_15_	5.2	704.1139	− 0.7	253.7	0.4	6	×	×	×	×	Yes	×	×	×	Yes
DCF-OH-Glc-GlcA	C_26_H_29_Cl_2_NO_14_	5.6	650.1049	1.6	233.7	0.0	3	×	×	×	×	×	Yes	×	×	×
DCF-OH-Glc-GlcA	C_26_H_29_Cl_2_NO_14_	4.2	650.1047	1.4	234.1	0.1	3	×	×	×	×	×	Yes	×	×	×
DCF-OH-Glc-Glc	C_26_H_31_Cl_2_NO_13_	4.1	636.1251	0.9	229.5	0.2	3	×	Yes	×	×	×	×	×	×	×
DCF-OH-Glc-Glc	C_26_H_31_Cl_2_NO_13_	3.0	636.1250	0.8	234.1	0.2	5	×	×	×	×	×	Yes	Yes	×	×
DCF-OH-Glc-Glc	C_26_H_31_Cl_2_NO_13_	3.0	636.1248	0.4	229.1	0.1	9	×	×	×	Yes	Yes	×	×	×	Yes
DCF-Glc-GlcA	C_26_H_29_Cl_2_NO_13_	5.3	634.1088	− 0.2	235.5	0.5	3	×	×	×	×	×	×	×	yes	×
DCF-Lac-OH-Glc-GlcA	C_26_H_27_Cl_2_NO_13_	4.1	632.0943	1.7	233.4	0.1	3	×	×	×	×	×	Yes	×	×	×
DCF-Glc-Glc	C_26_H_31_Cl_2_NO_12_	5.2	620.1299	0.5	232.1	0.1	3	×	Yes	×	×	×	×	×	×	×
DCF-Lac-OH-Glc-Glc	C_26_H_29_Cl_2_NO_12_	5.4	618.1140	0.0	229.7	0.6	18	×	Yes	×	Yes	Yes	Yes	Yes	Yes	×
DCF-Lac-Glc-Glc	C_26_H_29_Cl_2_NO_11_	5.4	602.1189	− 0.2	230.5	0.5	8	Yes	Yes	Yes	×	×	×	×	×	×
DCF-OH-Glc-Mal	C_23_H_23_Cl_2_NO_11_	5.7	560.0724	0.6	214.2	0.3	27	Yes	Yes	Yes	Yes	Yes	Yes	Yes	Yes	Yes
DCF-Glc-Mal	C_23_H_23_Cl_2_NO_10_	6.7	544.0770	− 0.4	211.6	0.3	23	Yes	Yes	Yes	Yes	Yes	Yes	Yes	Yes	×
DCF-Lac-OH-Glc-Mal	C_23_H_21_Cl_2_NO_10_	5.5	542.0618	0.5	210.8	0.2	24	Yes	×	Yes	Yes	Yes	Yes	Yes	Yes	Yes
DCF-diOH-Glc	C_20_H_21_Cl_2_NO_9_	3.5	490.0650	− 3.2	216.2	0.4	3	×	Yes	×	×	×	×	×	×	×
DCF-diOH-Glc	C_20_H_21_Cl_2_NO_9_	3.6	490.0670	0.7	212.4	0.1	12	×	×	×	Yes	Yes	Yes	Yes	×	×
DCF-OH-Glc	C_20_H_21_Cl_2_NO_8_	4.9	474.0720	0.7	211.4	0.2	27	Yes	Yes	Yes	Yes	Yes	Yes	Yes	Yes	Yes
DCF-Lac-OH-Glc	C_20_H_19_Cl_2_NO_7_	4.7	456.0614	0.7	197.5	0.2	18	×	×	×	Yes	Yes	Yes	Yes	Yes	Yes
DCF-OH	C_14_H_11_Cl_2_NO_3_	7.9	312.0191	0.6	164.4	0.1	27	Yes	Yes	Yes	Yes	Yes	Yes	Yes	Yes	Yes
DCF	C_14_H_11_Cl_2_NO_2_	8.6	296.0244	1.5	159.3	0.1	27	Yes	Yes	Yes	Yes	Yes	Yes	Yes	Yes	Yes
DCF-Lac-OH	C_14_H_9_Cl_2_NO_2_	7.6	294.0088	1.5	159.5	0.1	24	Yes	Yes	×	Yes	Yes	Yes	Yes	Yes	Yes
DCF-Lac	C_14_H_9_Cl_2_NO	7.6	278.0139	1.8	154.6	0.1	27	Yes	Yes	Yes	Yes	Yes	Yes	Yes	Yes	Yes

## Conclusions

A new powerful workflow for the detection of drug-related metabolites in plants upon uptake of the drug from the growing medium was developed. Analyzing extracts from nine different edible plants, grown hydroponically in a DCF-containing growing medium, allowed the listing of a range of new tentative DCF-related metabolites. This fact can be seen as proof for the usefulness of the new “reverse-engineering” workflow, combining information from DT measurements resulting in compound-specific ^DT^CCS_N2_ values respectively with data from chromatography and accurate mass measurements from MS.

The present work was primarily focused on qualitative analysis, i.e., the detection of tentative drug metabolites in plant matrices. However, as has been demonstrated in previous work [26], if quantitative or due to the lack of standards semi-quantitative analysis is desired, the proposed methodology could be seen as a first screening step, providing the information needed for setting up a multiple-reaction-monitoring method on a triple-quadrupole MS allowing the selective and sensitive analysis of the proposed substances. Future studies should also reveal whether this new approach will also be beneficial in other application fields.

## Abbreviations

DCF: Diclofenac
DCF-Lac: Diclofenac-lactam
DCF-OH: Hydroxylated diclofenac
DCF-Lac-OH: Hydroxylated diclofenac-lactam
Glc: Glucose
GlcA: Glucuronic acid
Mal: Malonic acid
